# Report of Cases

**Published:** 1869-07

**Authors:** Theo. N. Lescher

**Affiliations:** Register


### Report of Cases.

Under treatment in the Elmira Eye & Ear Institute, for the Quarter ending July 1st, 1869:

| | |
|---|---|
| Under treatment for Granular Lid | 10 |
| " " " Scrofulous Ophthalmia, | 5 |
| " " " Gonorrhœal, | 4 |
| " " " Glaucoma, | 1 |
| " " " Catarrhal Ophthalmia | 6 |
| " " " Simple Conjunctivitis, | 1[illegible] |
| " " " Suppurative Keratitis, | 4 |
| " " " Opacity of Cornea, | 4 |
| " " " Incised wound of Cornea, | 4 |
| " " " Otorrhœa, | 6 |
| " " " Deafness, | 4 |
| " " " Catarrh and Disease of Throat | 7 |
| " " " Rheumatic Iritis, | 1 |
| " " " Otalgia | 2 |
| " " " Spermatorrhoea, | 14 |
| Operated upon for Cataract, | 6 |
| " " " Iridectomy, | 2 |
| " " " Inversion of Eyelids, | 3 |
| " " " Pterygium, | 1 |
| " " " Strabismus, (Cross Eye,) | 2 |
| " " " Obstruction Lachrymal Duct, | 1 |
| " " " Cleft Palate, | 2 |
| " " " Removal of Tumors, | 3 |
| " " " Hœmorrhoids | 2 |
| " " " Amputation Scirrhus Breast, | 1 |
| " " " Hernia, | 1 |
| " " " Nasal Polypii, | 2 |
| " " " Epithelioma, lower lip, | 1 |
| " " " Excision lower jaw, | 1 |
| " " " New lip, (plastic operation,) | 1 |
| " " " Amputation of Thigh, | 1 |
| " " " Ruptured Perinæum | 2 |
| Insertion of Artificial Eyes, | 6 |
| Minor Surgical Operations, | 10 |
| Total number of Cases, | 136 |

THEO. N. LESCHER, Register.

